# Application of Nanotechnology in Detection and Removal of Pollutants in Water System

**DOI:** 10.3390/nano16181154

**Published:** 2026-09-14

**Authors:** Rajpreet Kaur, Mandeep Singh Bakshi

**Affiliations:** Department of Natural and Applied Sciences, University of Wisconsin-Green Bay, Green Bay, WI 54311, USA; bakshim@uwgb.edu

Access to clean and safe water remains a major environmental challenge due to the increasing release of organic pollutants, dyes, nutrients, microorganisms, and persistent contaminants into aquatic environments. Conventional treatment technologies may face limitations when dealing with complex and emerging contaminants, particularly at low concentrations. Nanomaterials have therefore attracted considerable attention because of their high surface area, tunable surface chemistry, catalytic activity, and multifunctionality [1]. The eight contributions included in this Special Issue highlight the diverse applications of engineered, functionalized, and green-synthesized nanomaterials for pollutant detection, adsorption, degradation, disinfection, and wastewater treatment.

In the context of this Editorial, the term “emerging nanomaterials” does not exclusively refer to newly discovered chemical compositions. Rather, it includes emerging material architectures, functionalization strategies, hybrid systems, green synthesis approaches, and novel application configurations. Thus, established materials such as silver (Ag) and zinc oxide (ZnO) nanoparticles are considered from the perspective of their specific treatment strategies or applications rather than chemical novelty alone. At the same time, newer platforms, including graphitic carbon nitride (g-C_3_N_4_), metal–organic frameworks (MOFs), covalent organic frameworks (COFs), MXenes, and hybrid materials, continue to broaden the possibilities for advanced water remediation.

**Advances in Nanomaterial-Enabled Water Treatment and Detection**

Two contributions demonstrate the potential of COF-based materials for environmental monitoring. Wei et al. developed a tris(4-formylphenyl)amine–4,4′,4″-(1,3,5-triazine-2,4,6-triyl)triphenylamine covalent organic framework (TFPA–TTA–COF) coating on gully-like stainless-steel fibers for the extraction of phenolic compounds, coupled with gas chromatography–flame ionization detection (GC-FID) [2]. Similarly, Yuan et al. developed a 1,3,5-tris(aminophenyl)benzene (TPB)–2,5-dimethoxyterephthalaldehyde (DMTP) covalent organic framework (TPB–DMTP COF)-coated fiber for the extraction of phthalic acid esters (PAEs) using solid-phase microextraction coupled with gas chromatography–flame ionization detection (SPME-GC-FID) [3]. Both systems demonstrated low detection limits and good durability over repeated extraction cycles, highlighting the importance of porous architectures, surface engineering, and robust material–substrate interactions for practical analytical applications.

Nanomaterials also demonstrate considerable potential for complex wastewater treatment. Abou-Okada et al. investigated Ag and ZnO nanoparticles for aquaculture wastewater treatment and reported high removal efficiencies for ammonia-nitrogen and other contaminants, with ZnO showing particularly effective reductions in chemical oxygen demand and microbial populations [4]. Importantly, combining Ag and ZnO did not improve performance and, in some cases, resulted in reduced efficiency because of aggregation-induced deactivation. This finding highlights an important challenge for multifunctional materials: increasing compositional complexity does not necessarily improve treatment performance. Hybrid nanomaterials should therefore be designed based on mechanistic complementarity and controlled interfacial interactions.

The potential of green-synthesized nanomaterials is demonstrated by Al-Ghamdi et al., who report a one-step biosynthesis of tungsten oxide (WO_3_) using *Kigelia pinnata* leaf extract for photocatalytic dye degradation [5]. The study supports the development of greener and solar-responsive photocatalytic systems, although future investigations should increasingly move beyond model pollutants and controlled laboratory conditions. Real wastewater constituents can significantly influence catalytic activity, while pollutant disappearance alone may not demonstrate complete remediation because potentially harmful transformation products can be generated.

The review contributions further emphasize the broad potential of nanomaterials for water disinfection and persistent organic pollutant removal through antimicrobial activity, adsorption, photocatalysis, redox processes, and magnetic recovery [1,6]. Adsorption-based approaches are represented by cellulose–activated carbon gels and functionalized CuFe_2_O_4_ nanoparticles, demonstrating the importance of combining removal efficiency with structural stability, recoverability, and reusability [7,8]. Collectively, the contributions of this Special Issue demonstrate that surface chemistry, morphology, active-site accessibility, and material stability are as important as composition in determining nanomaterial performance.

**Emerging Challenges and Future Perspectives**

Despite promising laboratory-scale results, the translation of nanomaterials into practical water treatment systems remains a major challenge. Most studies are still performed using synthetic single-contaminant solutions and batch experiments, whereas real water and wastewater contain complex mixtures of inorganic ions, natural organic matter, suspended solids, microorganisms, and competing pollutants. These components can alter nanoparticle aggregation, surface charge, adsorption behavior, and catalytic activity. Future research should therefore increasingly focus on real water matrices, continuous-flow and pilot-scale systems, and long-term operational performance. Stability, fouling, regeneration efficiency, material loss, and performance under fluctuating conditions should be evaluated alongside removal efficiency. In addition, technological feasibility should be assessed through techno-economic analysis (TEA) and life-cycle assessment (LCA), considering material synthesis, energy consumption, recovery, regeneration, and end-of-life management [9].

Environmental safety and sustainability also require greater attention. During synthesis, treatment, regeneration, or disposal, nanoparticles and dissolved components may potentially be released into the environment, where they can undergo aggregation, dissolution, oxidation, and surface transformation. These processes may alter their mobility, persistence, bioavailability, and toxicity [10]. The environmental fate and ecotoxicological implications of nanomaterials, together with transformation products generated during photocatalytic or oxidative treatment, should therefore be systematically evaluated. In this context, the Safe and Sustainable by Design (SSbD) approach is increasingly relevant because it integrates material performance, safety, resource efficiency, environmental impact, and end-of-life considerations from the early stages of material development [11].

Future research is also likely to expand beyond conventional metal and metal oxide nanoparticles. g-C_3_N_4_ offers opportunities for visible-light-driven photocatalysis, while MOFs and COFs provide highly tunable structures for adsorption, sensing, and catalytic applications [12,13]. MXenes and their composites have also emerged as promising materials for adsorption, membrane separation, and catalytic treatment, although challenges related to stability, oxidation, scalability, and environmental impact remain [14]. Hybrid materials combining complementary properties may provide improved functionality, but their increased complexity should be justified by measurable advantages over simpler alternatives. Data-driven approaches and machine learning may further support rational material design and process optimization; however, their success will depend on reliable and standardized datasets describing material properties, experimental conditions, and long-term treatment performance [15].

**Concluding Remarks**

The contributions assembled in this Special Issue demonstrate the diverse and expanding role of nanomaterials in water and wastewater management, ranging from contaminant detection and adsorption to photocatalytic degradation, disinfection, and complex wastewater treatment. However, the future of nanomaterial-enabled water treatment should not be defined solely by high removal efficiencies obtained under ideal laboratory conditions.

Successful translation will depend on whether materials can maintain their performance in real water matrices, operate continuously, be efficiently recovered and regenerated, minimize environmental risks, and remain economically and environmentally viable at larger scales. Thus, emerging nanomaterials should be understood not only through chemical novelty but also through advances in material architecture, functionalization, hybridization, sustainable synthesis, and safer practical implementation. The integration of SSbD principles, environmental risk assessment, LCA, TEA, and data-driven design will be increasingly important in moving promising nanotechnologies from laboratory studies toward real-world water treatment applications.
